# Experience with the Cardinal Coordinate System Contributes to the Precision of Cognitive Maps

**DOI:** 10.3389/fpsyg.2017.01166

**Published:** 2017-07-11

**Authors:** Xin Hao, Yi Huang, Yiying Song, Xiangzhen Kong, Jia Liu

**Affiliations:** ^1^State Key Laboratory of Cognitive Neuroscience and Learning, IDG/McGovern Institute for Brain Research, Beijing Normal University Beijing, China; ^2^Department of Psychology, Tsinghua University Beijing, China; ^3^Beijing Key Laboratory of Applied Experimental Psychology, School of Psychology, Beijing Normal University Beijing, China

**Keywords:** cardinal coordinate system, cognitive map, entorhinal cortex, hippocampus, voxel-based morphology, functional connectivity

## Abstract

The coordinate system has been proposed as a fundamental and cross-culturally used spatial representation, through which people code location and direction information in the environment. Here we provided direct evidence demonstrating that daily experience with the cardinal coordinate system (i.e., east, west, north, and south) contributed to the representation of cognitive maps. Behaviorally, we found that individuals who relied more on the cardinal coordinate system for daily navigation made smaller errors in an indoor pointing task, suggesting that the cardinal coordinate system is an important element of cognitive maps. Neurally, the extent to which individuals relied on the cardinal coordinate system was positively correlated with the gray matter volume of the entorhinal cortex, suggesting that the entorhinal cortex may serve as the neuroanatomical basis of coordinate-based navigation (the entorhinal coordinate area, ECA). Further analyses on the resting-state functional connectivity revealed that the intrinsic interaction between the ECA and two hippocampal sub-regions, the subiculum and cornu ammonis, might be linked with the representation precision of cognitive maps. In sum, our study reveals an association between daily experience with the cardinal coordinate system and cognitive maps, and suggests that the ECA works in collaboration with hippocampal sub-regions to represent cognitive maps.

## Introduction

Spatial cognition is a fundamental ability that humans rely on to interact with the environment. In this ability, coordinate systems play a pivotal role to connect scattered locations into a cognitive map (Burgess, 2006; Carpenter et al., 2015). For example, many people tended to use the cardinal coordinate system (i.e., east, west, north, and south) to label directions and locations (e.g., “five miles to the northwest"). However, little is known about whether and how the use of the cardinal coordinate system during daily navigation contributes to the representation of cognitive maps.

Previous behavioral studies indicated that people may adopt the cardinal coordinate system to organize their spatial memory. For example, participants perform better in pointing to landmarks in familiar environments when facing the north direction (Frankenstein et al., 2012) and learn new environments faster when they begin exploration with a northward facing direction (Gagnon et al., 2014), suggesting that cognitive maps have a principle orientation corresponding to the cardinal directions. However, direct evidence regarding whether daily experience of using the cardinal directions is beneficial for the representation of cognitive maps is still lacking. Given that the orientations of cognitive maps are determined at least partly by daily navigation experiences (Marchette et al., 2011; Frankenstein et al., 2012), here we hypothesized that individuals who rely more on the cardinal directions during daily navigation possessed better cognitive maps.

More importantly, the neural basis by which the cardinal coordinate system may contribute to cognitive maps has rarely been investigated. Firstly, the neural locus associated with daily experience of cardinal coordinate system has not yet been identified. Two lines of evidence at different levels converge to implicate the representation of the cardinal coordinate system in human brain. At the neuronal level, electrophysiological recording revealed grid-like spiking in the entorhinal cortex (EC) of humans (Jacobs et al., 2013), homologous to grid cells in rodents, with its constant spatial phase structure enabling a general and environmental-invariant representation of spatial map (Hafting et al., 2005; Krupic et al., 2015). And head-direction cells have been found in the rodent postsubiculum (Taube et al., 1990; Taube, 2007) and EC (Sargolini et al., 2006) responsible for directional information. At the regional level, Vass and Epstein (2013) found that activation patterns in the presubiculum can distinguish between the four cardinal facing directions. Further, Chadwick et al. (2015) showed that the EC and subicular region of the hippocampus code for cardinal goal directions as well as facing directions. Importantly, with prolonged experience, firing patterns of grid cells shift from a local map to a coherent global map (Carpenter et al., 2015), indicating a critical role of experience in forming a global spatial representation in the EC. Yet, no studies so far have examined where the daily experience of using the cardinal coordinate system is associated with the structure or function of human brain regions.

Secondly, it is unknown how the neural correlates of the cardinal coordinate system interact with other regions to represent cognitive maps. Besides grid cells and head direction cells, a variety types of cells have been revealed in the hippocampal formation in rodents that are responsible for different components of cognitive maps, such as place cells (O’Keefe and Dostrovsky, 1971) and boundary cells (Barry et al., 2006). Similar neural underpinnings have been identified in human brain (e.g., Ekstrom et al., 2003; Hassabis et al., 2009; Jacobs et al., 2013). These components need to be combined together to form cognitive maps (Knierim et al., 1995; Iaria et al., 2007), and the cardinal coordinate system may provide a reference frame for integrating them possibly through correlated spontaneous neural activity between regions responsible for different components.

To investigate whether and how the use of the cardinal coordinate system during daily navigation contributes to representation of cognitive maps, we asked a large number of participants (*N* = 284) to report their daily experiences of using the cardinal coordinate system, as Hegarty et al. (2002) did in their research. Then, we examined whether individuals who relied more on the cardinal coordinate system possessed better cognitive maps, with the representation precision of cognitive maps measured by an indoor pointing task (Bennett, 1996; Schinazi et al., 2013). To identify neuroanatomical correlates of daily experience with the cardinal coordinate system, we performed a whole brain voxel-based morphometry (VBM) analysis to correlate the gray matter volume (GMV) of each voxel across the brain with individuals’ reliance on the cardinal coordinate system. Finally, we used resting-state functional connectivity (rsFC) analysis to explore how the neural substrates of the cardinal coordinate system identified in the VBM analysis interact with other regions for better cognitive maps.

## Materials and Methods

### Participants

Two hundred and eighty-four college students (age range: 19–25; mean age = 21.6, *SD* = 1.03, 152 males) of Beijing Normal University, Beijing, China, participated in the study. None of the participants reported a history of neurological or psychiatric disorders. This study is part of an ongoing project (Gene Environment Brain and Behavior) (e.g., Huang et al., 2014; Wang et al., 2014; Kong et al., 2015; Song et al., 2015). All participants took part in the behavior task and structural MRI scan, among which 176 also participated in the resting-state fMRI (rs-fMRI) scan. All experiments and analysis methods were performed in accordance with the relevant guidelines and regulations of Beijing Normal University’s Institutional Review Board (Human Subjects Division), which approved all the experimental protocol and procedures. Written informed consent was obtained from each participant before the experiment.

### Behavior Tasks

#### Assessment of Daily Experience with the Cardinal Coordinate System

Participants were asked to explicitly rate the extent to which they relied on the cardinal coordinate system in their daily navigation, “I tend to think of my environment in terms of cardinal directions (north, south, east, and west).” This item is one of the fifteen items from the Santa Barbara Sense of Direction questionnaire (SBSOD) (Hegarty et al., 2002). The statement was ranked in a five-point Likert-type scale, as in the SBSOD. The score ranged from 1 to 5, consecutively indicating “strongly disagree” (the least experienced) to “strongly agree” (the most experienced). The assessment of daily experience with the coordinate system was performed in group on a separate day from the assessment of cognitive maps. In addition, the score of the question on the cardinal coordinate system highly correlated (*r* = 0.693, *p* < 0.001) with the score of the map factor of the SBSOD (Condon et al., 2015), indicating the validity of the item to assess participates’ use of the cardinal coordinate system.

In addition, to examine whether the cardinal coordinate system in humans is independent of language ability, we asked participants to evaluate the difficulty they had in comprehending complex verbal instructions as an index for their language ability, “Do you have difficulty in remembering complex verbal instructions? (ranging from 0 ‘very easy’ to 4 ‘very difficult’).”

#### Assessment of Cognitive Maps

An indoor landmark pointing task (Montello et al., 1999; Hegarty et al., 2002) was used to assess the precision of participants’ internal cognitive maps. The task took place inside an empty room in the campus with its windows and external cues covered. The accomplishment of the task requires representation of facing directions as well as knowledge of the landmarks regarding their locations, directions, and configurations; therefore the participants had to rely on their internal cognitive maps of the campus to point in the direction of the campus landmarks correctly. Participants were tested individually. After being taken into the room, the participant was asked to stand on a circular plastic dial (diameter, 2 m; measure unit, 1°; maximum scale, 360°) laid on the floor, with the heelpiece of the right foot right on the center point of the dial. Before the start of the experiment, the facing direction of the participant was calibrated by turning him/her to face the direction of the campus north gate, and then the participant was told that his/her facing direction was toward the campus’ north gate, which corresponded to zero degree on the dial (i.e., North). After finishing each trial, the participant resumed to face the north gate (i.e., the zero degree on the dial). For each trial, after being given the name of one landmark (e.g., a building or a statue) in the campus verbally, the participant had to point out the direction of the landmark by turning the facing direction from the north to the chosen direction. The measuring dial was placed on the floor and the participants were asked to stand on it to perform the pointing task. Thus, the scale of the degree that the participant’s right tiptoe pointed to on the dial was recorded as the response by an experimenter. The pointing error was calculated as the smallest absolute difference between the actual direction of the landmark and the participant’s response (range: 0–180°) for each trial, and the mean error averaged across all trials of a participant was used as the measurement of the precision of his/her cognitive map (i.e., larger error indicating less precision). Participants were not allowed to read the dial at any time and no feedback was given during the experiment. After the participants were instructed how to make pointing judgment and were aware of the importance of their feet’s position, we started the formal experiment. Eleven well-known campus landmarks in various directions were given (i.e., 11 trials in total). Note that all participants had lived in the campus for more than 2 years at the time of the test. Before the formal experiment, we guided all the participants to familiarize the position of the experimental room. In addition, the participants’ level of familiarity with the tested landmarks and the experimental building was collected after the pointing task. A pen-and-paper familiarity questionnaire comprised the 11 tested campus landmarks in the pointing task was administrated. Participants rated their degree of familiarity with each landmark on a scale ranging from 1 “very unfamiliar” to 7 “very familiar.” The mean score of all landmarks was used as an index of the level of familiarity with the landmarks for each participant.

### Data Acquisition

The structural MRI and rs-fMRI data were acquired on a Siemens 3T scanner (MAGENTOM Trio, a Tim system) with a 12-channel phased-array head coil at the BNU Imaging Center for Brain Research, Beijing, China. T1-weighted structure images were acquired with a magnetization-prepared rapid gradient-echo (MPRAGE) sequence (TR/TE/TI = 2530/3.45/1100 ms, FA = 7°, voxel size = 1 mm × 1 mm × 1 mm, slice thickness = 1.33 mm, number of volumes = 128) for each participant. In addition, for the rs-fMRI scanning, the participants were asked to close their eyes, remain awake, keep still, and not think about anything systematically. The rs-fMRI scanning lasted for 8 min. When the scan was finished, each participant was asked whether he/she had fallen asleep during the scan. Those who reported having fallen asleep were asked to complete the resting-state scan for a second time. T2^∗^-weighted functional images in resting state were acquired with a gradient-echo, echo-planar imaging (EPI) sequence (TR = 2000 ms, TE = 30 ms, FA = 90°, FOV = 200 mm × 200 mm, matrix = 64 × 64, number of slices = 33, voxel size = 3.125 mm × 3.125 mm × 3.6 mm, number of volumes = 240).

### Data-Analysis

#### VBM Analysis

Voxel-based morphometry was employed to quantify gray matter (GM) volume in each voxel across the whole brain (Ashburner and Friston, 2000). In this study, VBM was performed using SPM8 (Statistical Parametric Mapping, Wellcome Department of Imaging Neuroscience, London, United Kingdom) and DARTEL (Wellcome Department of Imaging Neuroscience). First, image quality was assessed by manual visual inspection. Second, the origin of the brain was manually set to the anterior commissure for each participant. Third, images were segmented into four distinct tissue classes: GM, white matter, cerebrospinal fluid and non-brain tissue (e.g., skull and scalp) using a unified segmentation approach (Ashburner and Friston, 2005). Forth, the segmented GM images were rigidly aligned and resampled to 2 mm × 2 mm × 2 mm. Fifth, the images were non-linearly registered with DARTEL, which involves iteratively computing a study-specific template based on the tissue maps from all participants and then warping all participants’ GM images into the generated template to increasingly improve the alignment (Ashburner, 2007). Sixth, the GM images for each participant were normalized to standard MNI space, and the GM voxel values were modulated by multiplying the Jacobian determinants derived from the normalization to preserve the volume of tissue from each structure after warping. The modulated GM images were then smoothed with an 8-mm full width at half maximum (FWHM) isotropic Gaussian kernel. Finally, to exclude boundary effects between gray matter and white matter, the modulated images were masked with an absolute threshold of 0.2. The masked modulated GM images were used for further statistical analyses.

#### Neuroanatomical Correlates of Daily Experience with the Cardinal Coordinate System

To investigate the neuroanatomical correlates of daily experience with the cardinal coordinate system, statistical analyses were performed using a general linear model (GLM). The statistical analysis treated the self-report reliance on the cardinal coordinate system in daily navigation as the variable of interest, the GM volume in each voxel as dependent variables, and gender, age, and total intracranial volume as confounding covariates. Multiple comparison correction was performed on statistical map using the 3dClustSim program implemented in AFNI^1^ (*version 16.1.13, 2016*). A threshold of cluster-level *p* < 0.005 and voxel-level *p* < 0.005 (cluster size > 194 voxels) was set based on Monte Carlo simulations in the whole brain mask.

#### rs-fMRI Data Preprocessing

For each participant, in order to get stable resting state data, the first four volumes were discarded. Then the remaining 236 volumes were preprocessed with FMRIB Software Library (FSL^2^), including head motion correction (by aligning each volume to the middle volume of the image with MCFLIRT), spatial smoothing (with a Gaussian kernel of 6-mm full-width half-maximum), intensity normalization, and the removal of linear trend. Then, a temporal band-pass filter (0.01–0.1 Hz) was applied with FSLMATHS to reduce low-frequency drifts and high-frequency noise.

Registration of each participant’s high-resolution anatomical image to a common stereotaxic space (the Montreal Neurological Institute 152-brain template with a resolution of 2 mm × 2 mm × 2 mm, MNI152) was accomplished using a two-step process (Andersson et al., 2007). First, a twelve-degrees-of-freedom linear affine was carried out with FLIRT. Second, the registration was further refined with FNIRT non-linear registration. Registration of each participant’s functional images to the high-resolution anatomical images was carried out with FLIRT to produce a six-degrees-of-freedom affine transformation matrix.

To eliminate physiological noise such as fluctuations caused by motion and cardiac and respiratory cycles, nuisance signals were regressed out as in previous studies (Fox et al., 2005; Biswal et al., 2010). Nuisance regressors included cerebrospinal fluid signal averaged from cerebrospinal fluid region, white matter signal averaged from white matter region, global signal averaged across the whole brain, six head realignment parameters obtained by rigid-body head motion correction, and the derivatives of each of these signals. The 4-D residual time series obtained after removing the nuisance covariates were registered to MNI152 standard space by applying the previously calculated transformation matrix.

#### rsFC between the EC and Hippocampal Sub-Regions

An ROI-based rsFC analysis was conducted to test connectivity between pre-selected ROIs. In addition, seed-based rsFCs with all voxels of hippocampal sub-regions were calculated, which were then correlated with behavioral performance. In the ROI-based rsFC analyses, the cluster identified with the VBM analysis that was associated with the cardinal coordinate system in the EC was taken as an ROI (i.e., the ECA). The ROIs of hippocampal sub-regions, including subiculum (Sub), cornu ammonis (CA), and dentate gyrus (DG), were derived as anatomic labels from the Juelich Histological Atlas implemented in FSL (**Figure 3**). To reduce the overlapping between the sub-regions maximally, the threshold for maximum probabilistic maps of the atlas was set at 80% and the remaining overlapped voxels were excluded from the rsFC analysis. The strength of the rsFC between two ROIs was estimated as the Pearson’s correlation between the mean time series of the ROIs.

To further examine the relation between rsFC and precision of cognitive maps, a voxel-wise seed-based rsFC analysis was performed with the ECA as the seed, and the rsFC of each voxel in the hippocampal sub-regions with the seed region was correlated with the pointing errors in the landmark pointing task, controlling for age and gender. Family wise error rate (FWE) in the FSL was set at *p* < 0.05 to correct for multiple comparisons within the masks of each hippocampal sub-region, respectively. All correlational coefficients were Fisher *z*-transformed, and the *z*-score was used in further analyses.

#### rsFC between the EC and the Whole Brain

In addition, the seed-based rsFC analysis was also performed at the whole-brain level, with the rsFC of each voxel across the whole brain with the ECA correlating with the pointing errors in the landmark pointing task. A threshold of cluster-level *p* < 0.05 and voxel-level *p* < 0.005 (cluster size > 141 voxels) was set based on Monte Carlo simulations in the whole brain mask.

## Results

**FIGURE 1:**
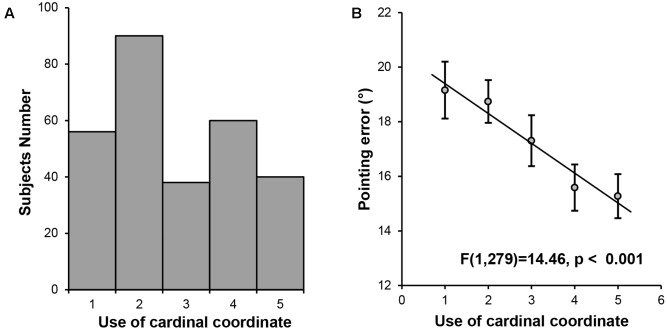
Behavioral results. **(A)** Histogram of the self-reported daily experience with the cardinal coordinate system. **(B)** A linear trend analysis on pointing error in the landmark pointing task among the five groups of participants defined by their daily experience with the cardinal coordinate system during navigation.

A significant amount of individual differences were found among the participants in the use of the cardinal coordinate system for daily navigation (Mean = 2.77, *SD* = 1.348). To establish the link between the cardinal coordinate system and cognitive maps, we investigated whether the extent to which the participants relied on the cardinal coordinate system predicted the precision of their internal cognitive maps. The indoor landmark pointing task was used to measure the precision of cognitive maps, with the pointing error calculated as the averaged smallest absolute differences between the participant’s response and the actual direction. A one-way ANOVA was performed on the mean pointing error among the five groups of participants defined by their preference for using the cardinal coordinate system in navigation. We found a significant main effect of group [*F*(4,279) = 4.16, *p* = 0.003], with a significant linear trend [*F*(1,279) = 14.46, *p* < 0.001, **Figure 1**], showing that individuals who relied more on the cardinal coordinate system in their daily navigation might possess more precise cognitive maps.

However, two confounding factors may account for the association between the coordinate system and cognitive maps. First, the participants had different levels of familiarity to the landmarks tested. Being familiar with specific landmarks means that one has increased knowledge concerning locations of the landmarks relative to unfamiliar landmarks (Thorndyke and Hayes-Roth, 1982), and thus, familiarity with the landmarks was likely to affect the precision of cognition maps. To rule out this possibility, we asked the participants to evaluate their level of familiarity to the landmarks tested after the pointing task. As expected, all participants reported that they were quite familiar with the landmarks since they had lived on the campus for at least 2 years. No score on familiarity was below 4 point in a 7-point scale. Importantly, the correlation between the familiarity score and the pointing error was not significant (*r* = -0.04, *p* = 0.50), suggesting that individual differences in the precision of cognitive maps were not affected by participants’ familiarity with the campus. Further, after regressing out the familiarity score from the pointing error, the one-way ANOVA analysis among the five groups showed the same pattern [main effect of group, *F*(4,279) = 4.04, *p* = 0.004; linear trend, *F*(1,279) = 13.76, *p* < 0.001]. Thus, the association between the coordinate system and cognitive maps was unlikely to be influenced by how familiar the participants were with the landmarks.

Second, because the use of the cardinal coordinate system in humans requires the implementation of language (i.e., east, west, south, and north), it is possible that individuals with better language ability are better at using the cardinal coordinate system. Here we examined whether the use of the cardinal coordinate system in our participants was independent of their language ability. To do this, we asked participants to evaluate the difficulty in comprehending complex verbal instructions as an index for their language ability. First, one-way ANOVA on the self-reported language ability scores revealed no significant main effect of group [*F*(4,279) < 1] or linear trend [*F*(1,279) < 1], suggesting that individual differences in daily experience with the cardinal coordinate system were not related to participants’ language ability. Second, after regressing out the scores of language ability, the one-way ANOVA on pointing error revealed the same pattern [main effect of group, *F*(4,279) = 4.04, *p* = 0.004; linear trend, *F*(1,279) = 13.76, *p* < 0.001], suggesting that the relation between the cardinal coordinate system and cognitive maps was independent of participants’ language ability. Taken together, we observed a link between the cardinal coordinate system and cognitive maps. Next we explored the neuroanatomical basis underlying the daily experience with the cardinal coordinate system in the human brain, and then characterized its role in interacting with other navigation-related regions to form cognitive maps.

**FIGURE 2:**
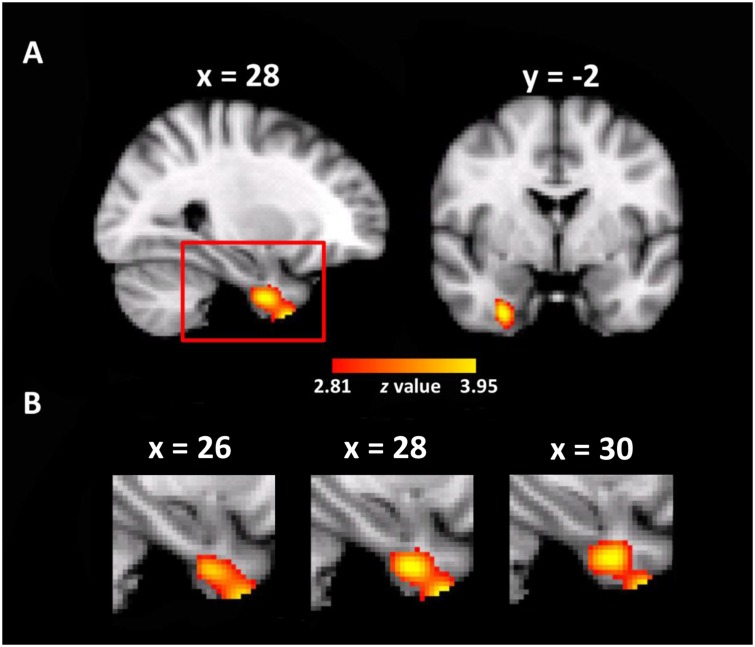
Neuroanatomical correlates of daily experience with the cardinal coordinate system. **(A)** A cluster where the gray matter volume of each voxel positively correlated with daily experience with the cardinal coordinate in the entorhinal coordinate cortex (ECA) is shown in the MNI space (*p* < 0.005, whole-brain correction). **(B)** The enlarged views of the red box in different sagittal planes.

The whole brain VBM analysis revealed that the GM volume of a cluster in the right entorhinal cortex was significantly correlated with the use of the cardinal coordinate system in daily navigation (MNI coordinate of peak voxel: *x* = 28, *y* = -2, *z* = -40, *z*-value = 4.00, 212 voxels, *p* < 0.005, corrected, **Figure 2**). As expected, one-way ANOVA on the mean GM volume of this cluster showed a significant main effect of group [*F*(4,279) = 8.80, *p* < 0.001], as well as a significant linear trend [*F*(1,279) = 30.64, *p* < 0.001, **Figure 2**], suggesting that individuals who relied more on the cardinal coordinate system for daily navigation linked with larger GM volume in the right EC. No significant cluster was obtained in the left EC. For simplicity, the cluster in the EC is coined as the entorhinal coordinate area (ECA). Besides the ECA, there was another cluster in the right premotor cortex that also showed significant correlation with the use of the cardinal coordinate system (MNI coordinate of peak voxel: *x* = 48, *y* = -12, *z* = 56, *z*-value = 3.74, 371 voxels, *p* < 0.005, corrected). Finally, a cluster in the left inferior parietal lobe (IPL) was identified (MNI coordinate of peak voxel: *x* = -38, *y* = -82, *z* = 22, *z*-value = 3.47, 139 voxels), though it did not survive the multiple comparison correction. As the EC has been implicated in representing the coordinate system in previous studies (e.g., Jacobs et al., 2013), we focused on examining how the ECA interacted with the hippocampus that is the primary region consisting of a variety of navigation-related neurons to form cognitive maps.

**FIGURE 3:**
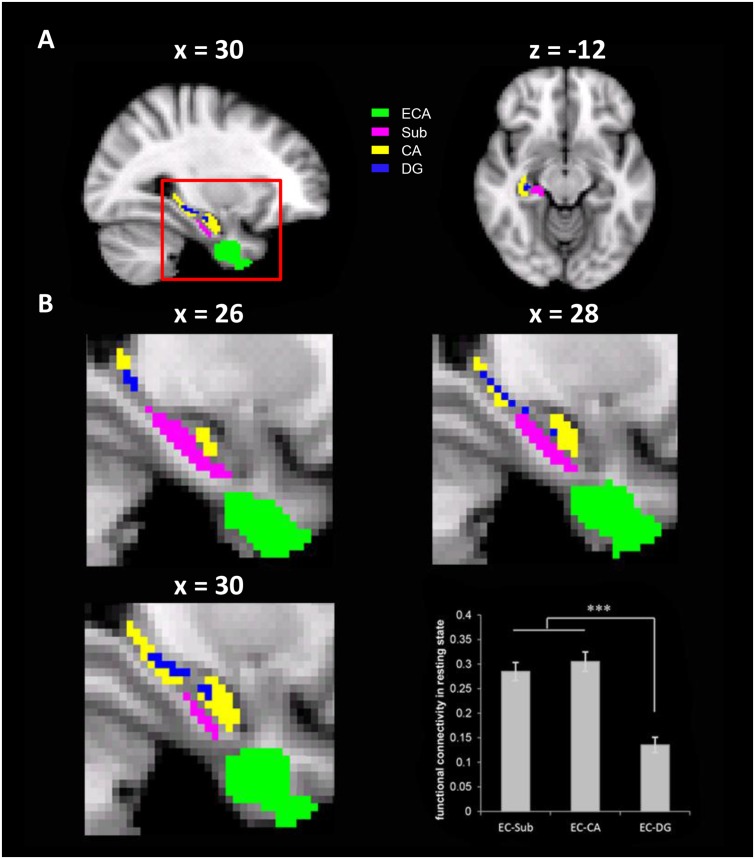
The resting state functional connectivity (rsFC) between the ECA and three anatomically defined sub-regions of the hippocampus. **(A)** The ECA and three anatomically defined sub-regions of the hippocampus. **(B)** The enlarged views of the red box in different sagittal planes. The bar chart showed the rsFCs between the ECA and the hippocampus sub-regions. ECA, entorhinal coordinate area; Sub, subiculum; CA, cornu ammonis; DG, dentate gyrus. ^∗∗∗^*p* < 0.001.

To do this, we performed ROI-based rsFC analyses on spontaneous fluctuation between the ECA and three anatomically defined hippocampal formation sub-regions (Sub, CA, and DG) in the right hemisphere, because the ECA was mainly localized in the right EC. Significant rsFC was found between the ECA and all three sub-regions [*t*_EC-Sub_ (175) = 15.49, *p* < 0.001; *t*_EC-CA_ (175) = 15.41, *p <* 0.001; *t*_EC-DG_ (175) = 8.61, *p* < 0.001, **Figure 3**], with the rsFC of ECA-Sub [*t*(175) = 10.55, *p* < 0.001] and the rsFC of ECA-CA [*t*(175) = 12.12, *p* < 0.001] significantly higher than the rsFC of ECA-DG. No significant difference was found between the rsFC of ECA-Sub and that of ECA-CA [*t*(175) = -1.53, *p* = 0.13]. Since anatomical distances among regions might affect the magnitude of the rsFCs, we normalized the magnitudes of the rsFCs by dividing the anatomical distances for each participant, and found the same pattern of results [EC-CA vs. EC-DG, *t*(175) = 12.98, *p* < 0.001; EC-DG vs. EC-DG, *t*(175) = 13.03, *p* < 0.001; EC-sub vs. EC-CA, *t*(175) = 1.75, *p* = 0.082]. Taken together, the significant rsFCs between the ECA and hippocampal sub-regions suggest that they are likely form a functional network possibly involved in the formation of cognitive maps.

**FIGURE 4:**
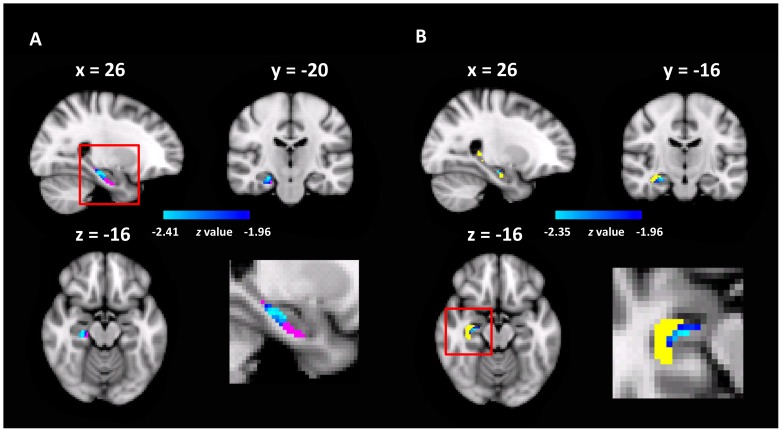
The hippocampal sub-regions where the rsFC with the ECA correlated with pointing errors (*p* < 0.05, FWE correction). **(A)** The Sub was shown in red, and the heat maps showed the cluster found in the Sub with the rsFC analysis; the enlarged view of the red box was shown in bottom right. **(B)** The CA was shown in yellow, and the heat maps showed the cluster found in the CA.

To test this hypothesis, we examined the functional significance of the rsFCs between the ECA and hippocampal sub-regions by correlating them with representation precision of cognitive maps. Specifically, a seed-based rsFC analysis was performed with the ECA as the seed. The rsFC of each voxel in the hippocampal sub-regions with the seed were correlated with the pointing errors in the landmark pointing task. We found that the rsFCs between the ECA and two clusters, one in the Sub (MNI coordinate of peak voxel: *x* = 26, *y* = -20, *z* = -16, *z*-value = 2.47, 37 voxels, *p* < 0.05, corrected, **Figure 4A**) and the other in the CA (MNI coordinate of peak voxel: *x* = 26, *y* = -16, *z* = -16, *z*-value = 2.35, 9 voxels, *p* < 0.05, corrected, **Figure 4B**), were negatively correlated with the errors in pointing the campus landmarks. That is, participants who had a stronger rsFC between ECA and Sub/CA were associated with higher accuracy in pointing the direction of the landmarks (i.e., higher precision of cognitive maps). No significant positive correlations were found. Therefore, the more closely the ECA and Sub/CA collaborate, the better the participant’s cognitive map representation might be.

To investigate whether the rsFCs of the ECA with other regions besides the hippocampus across the brain were related to pointing errors, we performed a whole brain seed-based rsFC analysis and found that the rsFC between the right middle frontal gyrus/frontal pole and the ECA was negatively correlated with pointing errors (MNI coordinate of peak voxel: *x* = 36, *y* = 34, *z* = 42, *z*-value = -3.81, 321 voxels, *p* < 0.05, corrected). No positive correlations were found. These results indicated that successful pointing might also require substantial cooperation between the ECA and the prefrontal cortex, which has long been recognized to play an important role in goal-directed navigation and re-planning of paths via top-down modulations (Maguire et al., 1998; Hartley et al., 2003; Spiers and Maguire, 2006; Brown et al., 2016).

## Discussion

In daily life, humans rely on coordinate systems to represent and organize spatial information in the environment. In the current study, we investigated whether and how daily experience with the cardinal coordinate system contributed to the representation of cognitive maps. Behaviorally, we found that individuals who relied more on the cardinal coordinate system for daily navigation associated with more precise cognitive maps, suggesting that the cardinal coordinate system is an important element for cognitive map. Neurally, we found that the GM volume of the ECA was positively correlated with participants’ daily experience with the cardinal coordinate system. Further, the FCs between the ECA and hippocampal sub-regions of CA/Sub might be linked with the representation precision of cognitive maps. In sum, these findings suggest that intrinsic interaction between the ECA and hippocampal sub-regions may underlie the contribution of the cardinal coordinate system to cognitive maps.

Our study revealed an association between daily experience with the cardinal coordinate system and the representation precision of cognitive maps. This result is congruent with previous behavioral studies showing that north-facing direction benefits performance when pointing targets of familiar environment as well as when learning novel environment (Frankenstein et al., 2012; Gagnon et al., 2014), suggesting that the orientations of cognitive maps are related to the cardinal coordinate systems. Our result extended these studies and provided clear evidence for the role of the cardinal coordinate system in cognitive maps by showing that daily experience with the cardinal coordinate system was indeed helpful in the representation of cognitive maps. More generally, this finding corroborated previous evidence suggesting that global coordinate systems were a contributing factor to cognitive maps (Burgess, 2006; Frankenstein et al., 2012; Gagnon et al., 2014). Why would the use of the cardinal coordinate system be critical for the representation of cognitive maps? Firstly, the coordinate system supports a scaffold containing multiple spatial relationships among elements in the environment that helps to combine discrete components of cognitive maps together (e.g., locations, facing directions) (McNamara, 1986). Secondly, the cardinal coordinate system, like all allocentric reference frames, provides stable spatial representation that is tolerant to individual’s constant changes of location, viewpoints, or heading direction in a complex environment (Burgess, 2006). In short, the cardinal coordinate system is likely to provide a reliable and stable scaffold that could group together discrete components into precise cognitive maps.

Neurally, we found that the GM volume of the right ECA was positively correlated with daily experience with the cardinal coordinate system. The ECA identified in our study located very close to the cluster found by comparing good vs. bad navigators grouped through the SBSOD (Wegman et al., 2014). Because our study focused on the cardinal coordinate system, one specific element of general spatial ability measured with SBSOD, it is reasonable that the ECA was also associated with general spatial ability. That is, good navigators may rely more on using the cardinal coordinate than bad navigators for successful navigation. Importantly, this result is in line with previous studies that revealed grid and conjunctive grid cells in the EC of rodents (Hafting et al., 2005; Fyhn et al., 2007) and humans (Doeller et al., 2010; Jacobs et al., 2013), which allows the representation and updating of spatial information with a generalized and universal spatial map (Hafting et al., 2005; Fyhn et al., 2007). In addition, activation patterns in the EC in human brain can distinguish between the cardinal directions (Chadwick et al., 2015). Besides, the head direction cells found in the EC may also be involved in representing the cardinal directions in the EC (Jacobs et al., 2010). These findings converge to indicate that the EC might support a representation of global reference frames such as the cardinal coordinate system during navigation. Moreover, given that prolonged experience plays a critical role in forming a representation of global reference frame in the EC (Carpenter et al., 2015), it is possible that individual differences in daily experience with the cardinal coordinate system may induce plasticity of neuronal activity in the ECA that cumulates in different alteration of the ECA neuronal morphology among individuals (Maguire et al., 2000; Frank et al., 2002; Draganski et al., 2004; Witter and Moser, 2006). Interestingly, while our study showed that the EC may be involved in the representation of the cardinal coordinate system that is a global reference frame, a recent study has revealed that the retrosplenial complex (RSC) codes the spatial representation in a reference frame anchored relative to local environmental landmarks (Marchette et al., 2014). That is, there may be functional division of labor between the EC and RSC in representing global and local reference frames, respectively.

Further, we found that the FC between the ECA and hippocampal sub-regions might contribute to precise representation of cognitive maps, corroborating the behavioral association between the cardinal coordinate system and cognitive maps and possibly providing neural basis for this association. This result fits nicely with previous studies showing that the EC serves as a key contributor to the signals in the hippocampus (Kloosterman et al., 2003; Witter and Moser, 2006), with grid cells in the EC providing the most abundant input to place cells and head direction cells in the hippocampus (Brun et al., 2008; Zhang et al., 2013; Hartley et al., 2014; Zhang et al., 2014). Thus, the EC and hippocampus may work collabrately during daily navigation. Therefore, the co-activation history between the EC and hippocampus during daily navigation may modulate intrinsic connectivity between them, and they may maintain meaningful interaction even during resting-state for precise representation of the cognitive maps and for better performance during navigation (Stevens et al., 2010). More specifically, though abundant synapse projections exist between the EC and all three sub-regions of the hippocampus, neurons in the EC provide significantly more inputs to the CA and Sub than to the DG (Hartley et al., 2014; Witter et al., 2014). In addition, electrophysiological recordings identified different distributions of navigation-related cells in the hippocampal sub-regions. That is, CA (especially CA1 and CA3) contained a high density of place cells, and Sub contained most head direction cells (Jung et al., 1994; Taube, 2007), whereas poor density of spatial cells has been found in DG. Taken together, this may explained the reasons why the strength of the ECA-CA/Sub FCs was higher than that of the ECA-DG FC, and why only the ECA-CA/Sub FCs, but not the ECA-DG FC, were associated the precision of cognitive maps. Notably, numerous studies have indicated that CA and Sub serve distinct functional roles in navigation, with the CA (especially CA1) encoding allocentric spatial relationships when learning novel environment (Leutgeb et al., 2005; Brun et al., 2008; Suthana et al., 2009), whereas the Sub was involved in retrieval of learned spatial information (Suthana et al., 2011). Therefore, we speculate that the FC of ECA-CA may underlie the contribution of the cardinal coordinate system in the construction of cognitive maps through encoding novel allocentric spatial relationships, whereas the FC of ECA-Sub may be helpful for more precise retrieval of spatial information from constructed cognitive maps via the cardinal coordinate system.

## Conclusion

Our study demonstrates the clear association between daily experience with the cardinal coordinate system and representation precision of cognitive maps, and shows that interaction between the ECA and hippocampal sub-regions may underlie this association. Several unaddressed issues are important topics for future research. First, the correlational nature of our findings does not permit inferences about the causal relationship between the cardinal coordinate system and cognitive maps. However, it is worthy of examining with well-controlled training experiments whether we can improve precision of our cognitive maps and promote our navigation abilities by increasing the use of cardinal coordinate system in future studies. Second, in addition to the EC and hippocampus, previous studies have revealed other regions involved in the representation of spatial information, such as the RSC coding for local reference frames (Marchette et al., 2014). While we focused on the interaction between the EC and hippocampus in this study, future studies examining interactions among other navigation-related regions are needed to understand more about how spatial representations are organized by integrating global and local reference frames. Third, the individuals who perform successfully in the pointing task may use a combination of survey, route, and landmark strategies; therefore, future studies are needed to measure individuals’ preferences for each specific mental representation of spaces, such as by a series of local maps, landmark-based navigation, and egocentric/allocentric maps, to reveal cortical regions may be involved in each aspect of cognitive maps and investigate the relationship between these spatial strategies and performance in pointing tasks. Finally, the use of the cardinal coordinate system was measured by self-reports in our study; future studies with more objective measures of the use of the cardinal coordinate system and more complex and widespread orientation tasks (e.g., Frankenstein et al., 2012; Marchette et al., 2014) are needed to further validate the association between the cardinal coordinate system and cognitive maps observed here.

## Author Contributions

XH, YH, and JL designed the experiments. XH, YH, YS, and XK conducted the experiments. XH and YH analyzed the data. XH, YS, and JL wrote the manuscript. JL supervised the project.

## Conflict of Interest Statement

The authors declare that the research was conducted in the absence of any commercial or financial relationships that could be construed as a potential conflict of interest.

## Footnotes

1 http://afni.nimh.nih.gov

2 www.fmrib.ox.ac.uk/fsl/
